# Facial Vibrotactile Stimulation Activates the Parasympathetic Nervous System: Study of Salivary Secretion, Heart Rate, Pupillary Reflex, and Functional Near-Infrared Spectroscopy Activity

**DOI:** 10.1155/2014/910812

**Published:** 2014-01-08

**Authors:** Hisao Hiraba, Motoharu Inoue, Kanako Gora, Takako Sato, Satoshi Nishimura, Masaru Yamaoka, Ayano Kumakura, Shinya Ono, Hirotugu Wakasa, Enri Nakayama, Kimiko Abe, Koichiro Ueda

**Affiliations:** ^1^Department of Dysphasia Rehabilitation, Nihon University of Dentistry, 1-8-13 Kanda-surugadai, Chiyoda-ku, Tokyo 101-8310, Japan; ^2^Department of 1st Oral and Maxillofacial Surgery, Nihon University of Dentistry, 1-8-13 Kanda-surugadai, Chiyoda-ku, Tokyo 101-8310, Japan; ^3^Department of Physics, Nihon University of Dentistry, 1-8-13 Kanda-surugadai, Chiyoda-ku, Tokyo 101-8310, Japan

## Abstract

We previously found that the greatest salivation response in healthy human subjects is produced by facial vibrotactile stimulation of 89 Hz frequency with 1.9 **μ**m amplitude (89 Hz-S), as reported by Hiraba et al. (2012, 20011, and 2008). We assessed relationships between the blood flow to brain via functional near-infrared spectroscopy (fNIRS) in the frontal cortex and autonomic parameters. We used the heart rate (HRV: heart rate variability analysis in RR intervals), pupil reflex, and salivation as parameters, but the interrelation between each parameter and fNIRS measures remains unknown. We were to investigate the relationship in response to established paradigms using simultaneously each parameter-fNIRS recording in healthy human subjects. Analysis of fNIRS was examined by a comparison of various values between before and after various stimuli (89 Hz-S, 114 Hz-S, listen to classic music, and “Ahh” vocalization). We confirmed that vibrotactile stimulation (89 Hz) of the parotid glands led to the greatest salivation, greatest increase in heart rate variability, and the most constricted pupils. Furthermore, there were almost no detectable differences between fNIRS during 89 Hz-S and fNIRS during listening to classical music of fans. Thus, vibrotactile stimulation of 89 Hz seems to evoke parasympathetic activity.

## 1. Background

In previous work, we created a vibrotactile stimulation apparatus to relax facial muscles and alleviate muscle stiffness after orofacial surgery. However, the apparatus proved to elicit salivation [1–3]. Considering that parasympathetic activity increases salivation but also has other effects, including changes in heart rate, heart rate variability (HRV), and pupil constriction [4], we investigated whether our device might have an effect on the parasympathetic nervous system in general.

To induce salivation is to combat xerostomia/dry mouth. Artificial saliva, humectants, and/or salivary gland massage can be used as a cure [5]. Although salivary gland massage may reinvigorate weak glands, leading to a more permanent solution, it can be difficult for people with disabilities to perform, and the efficacy of the treatment varies widely, depending on massaging skill. Furthermore, it is difficult to massage facial skin for more than 5 min due to fatigue except for the professional massager. In particular, Desmedt and godaux [6] firstly reported that the relaxation of masticatory muscles is evoked at a frequency of 80 Hz, but we have conducted tests on inducing salivation via vibrotactile stimulation using this alike frequency [2]. Salivation following vibrotactile stimulation of the face might be a response of the parasympathetic nervous system [1]. In that study, stimulation at a frequency of 89 Hz and an amplitude of 1.9 *μ*m using a single motor was the most effective for increasing salivation and showed no adaptation with continued daily use, as shown in the previous paper [1]. Other effects of parasympathetic stimulation include a decrease in heart rate and constriction of the pupils, whereas sympathetic stimulation has the opposite effects. Furthermore, the salivary glands secrete a copious amount of serous saliva during parasympathetic activity, whereas a more viscous secretion is released during sympathetic activity. In the present study, we investigated whether the 89 Hz-S stimulation described above may elicit parasympathetic activity by comparing activity in the brain as assessed by functional near-infrared spectroscopy (fNIRS) to heart rate, pupillary condition, and salivation during various stimuli.

On the other hand, when neuron was activated, the adjacent vessel in the region increases in blood flow by extension of vessels. Thus, a dilation of capillary increases an amount of blood in the organ and transmutes coefficient of oxidation reduction. However, a detailed mechanism is unclear, the phenomenon is used as the index of neuronal activity. Even so, PET, SPFCT, fMRI, and NIRS are provided evidence as a standard method of measurement. On the other hand, there is report that an astrocytic activity may be related to vasodepressor responses, recently [7]. Our research is performed by these actual achievements between the fNIRS and neuronal activity.

## 2. Study Design and Methods

**Figure 1:**
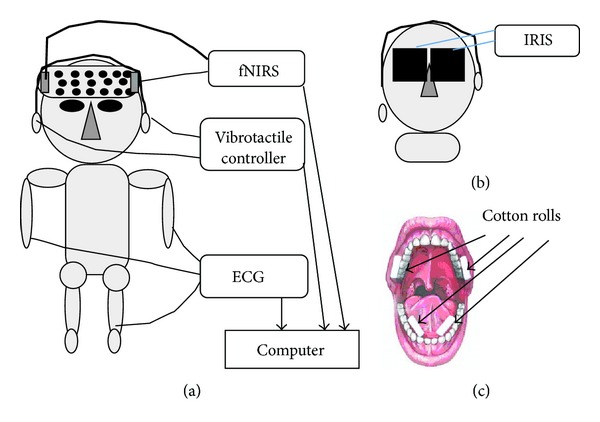
Experimental pattern diagrams. (a) Recording of fNIRS and heart beat during vibrotactile stimulation. (b) Recording of pupillary reflex during vibrotactile stimulation. (c) Cotton-roll method for recording of salivation.

We indicate experimental schemata of Figures 1(a), 1(b), and 1(c). Recording of fNIRS (functional near-infrared spectroscopy: OEG16 instrument, Spectratech Inc., Shelton, CT, USA) and heartbeat (ECG, electrocardiogram: HRV module, AD Instruments, Tokyo, Japan) during vibrotactile stimulation was shown in Figure 1(a). Recording of pupillary reflex using the IRIS device (Iriscorder, Hamamatsu Photonics Co., Tokyo, Japan) during vibrotactile stimulation was indicated in Figure 1(b). Cotton-roll method for recording of salivation was exhibited in Figure 1(c). We determined the amount of salivation using a dental cotton roll (1 cm across, 3 cm length) positioned at the opening of the secretory ducts (right and left sides of the parotid glands and right and left sides of the submandibular and sublingual glands).

### 2.1. Normal Subjects

Although we tried to collect the same subjects, we could not produce an exact same subject. However, subjects in each experiment are different, because each experiment was conducted on the different day. On the other hand, we included that about 50% of subjects were the same. We unified the whole of experimental items (salivation, pupil reflex and heart rate, HRV). In particular, experimental items of salivation and heart rate are the same.

This study was expended about one year. In Japan, there is difference in air temperature in the four seasons. We think when it was cold or hot, the heart rate will change. For this reason, we carried out the examination at a temperature-controlled room. Furthermore, we carried out the examination at the same time and place.

### 2.2. Vibrotactile Stimulation of the Face

The vibrotactile stimulation apparatus consists of an oscillating body and a control unit, described in detail in Hiraba et al. [3] and Lee et al. [8]. This apparatus elicits salivation, especially at the settings described above, when applied to either the parotid or submandibular gland, and daily use does not lead to adaptation [1].

**Figure 2:**
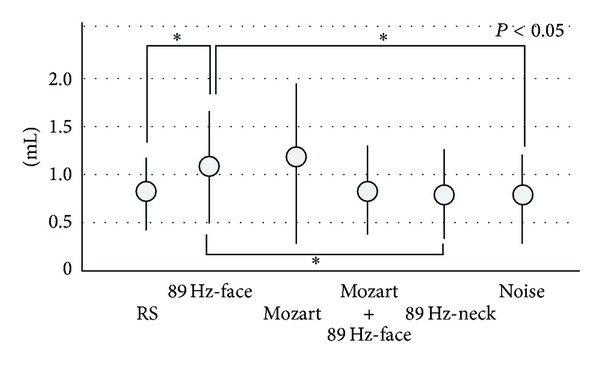
Total salivation from the parotid, submandibular, and sublingual glands after facial skin vibrotactile stimulation on the parotid glands. The increased salivation between RS (resting state) and 89 Hz-face (89 Hz-S on the face) was significant (paired *t*-test, *P* < 0.05), as it was between 89 Hz-face and 89 Hz-neck (89 Hz-S on nap of the neck), or Noise (listening to noise). “Mozart + 89 Hz-face” indicates listening and vibrating to Mozart and 89 Hz-face simultaneously.

We used the device to stimulate the faces (hereafter, 89 Hz-face) of subjects in this study. Furthermore, 114 Hz-face, classic music (Mozart, Eine kleine Nachtmusik), and noise were employed as contrast stimulus of 89 Hz-face. We measured total salivation from six glands (the right and left parotid glands and the right and left submandibular and sublingual glands) using the cotton-roll method, as shown in Figure 1(c) [1–3]. The results were compared to total salivation under the following conditions: in a resting state (RS), while listening to Mozart (Classic music: Mozart); Mozart + 89 Hz-face, while listening to noise (Noise) for 3 min; and during stimulation of the nap of the neck (89 Hz-neck). Listening to classical Mozart (Eine kleine Nachtmusik) tends to produce a relaxing feeling, and massaging the nape of the neck helps relax muscle tension. Thus, we expected Mozart + 89 Hz-face to have a synergistic relaxing effect, but Mozart + 89 Hz-neck was decided by the deletion of this data for the analysis difficulty after experiments. In other words, first aim of our experiment is the increase of salivation. Although HRV value of 89 Hz-neck is the largest, the increase of salivation of 89 Hz-neck is far less than the other stimuli, as shown in Figure 2. So we deleted data of Mozart + 89 Hz-neck from this cause.

### 2.3. Heart Rate during Vibrotactile Stimulation

**Figure 3:**
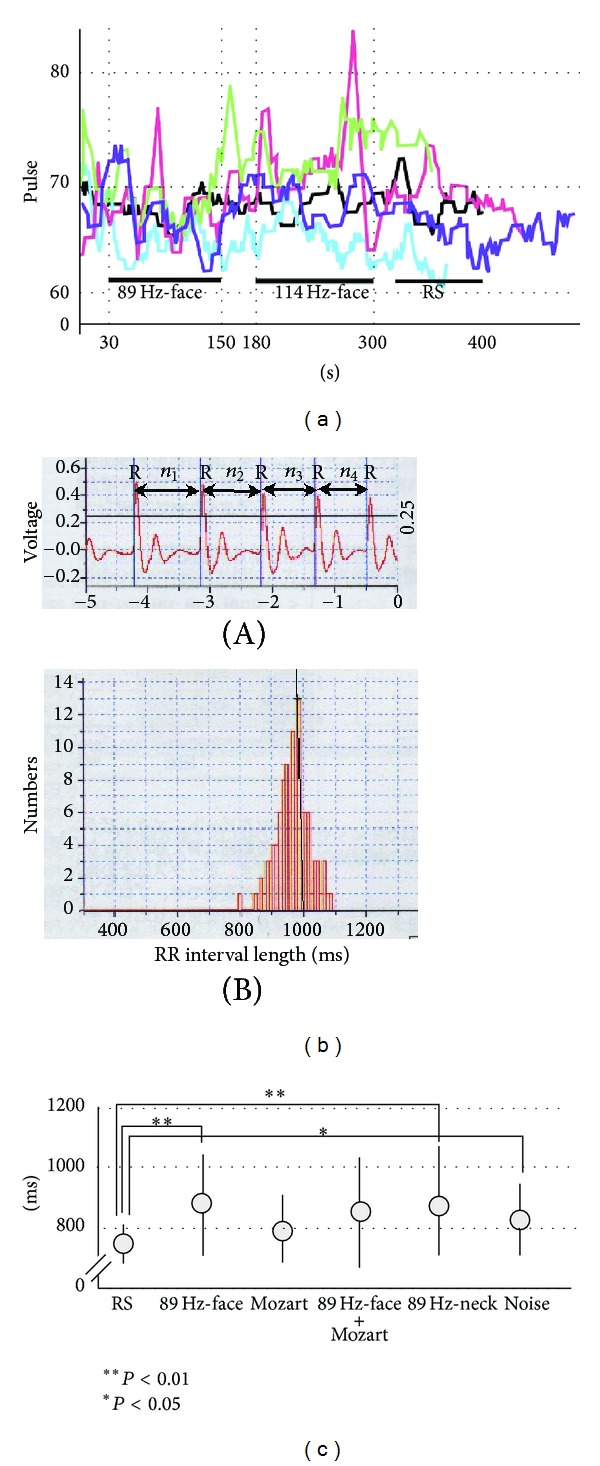
(a) Changes in pulse frequency during 89 Hz-S on the face (89 Hz-face), 114 Hz-S on the face (114 Hz-face), and resting stage. We assessed changes in pulse frequencies for five typical subjects. Note that all subjects showed decreased pulse frequency during 89 Hz-face. (b) HRV module analysis. Method used to measure RR intervals (*n*_1_, *n*_2_, *n*_3_, *n*_4_, etc.) on ECG recordings (B-a) and frequency spectrum based on RR interval length over 3 min during 89 Hz-face (B-b). Horizontal line indicates RR interval (ms), and vertical line indicates number. Note that the peak frequency spectrum was 1000 ms in this experiment, as shown in B-b. (c) Changes in peak frequency spectra of HRV modulation during various stimuli. We indicate 89 Hz-face, Mozart, Mozart + 89 Hz-face, 89 Hz-neck, and Noise, respectively. There were significant differences between RS and 89 Hz-face (paired *t*-test, *P* < 0.01), between RS and Mozart + 89 Hz-face (paired *t*-test, *P* < 0.01), and between RS and noise (paired *t*-test, *P* < 0.05).

We recorded changes in heart rate (pulse frequency) (P225F, Nihon-Kohden Co., Tokyo, Japan) of 10 normal subjects (6 males and 4 females; average age, 22 years) during vibrotactile stimuli of 89 Hz-face and 114 Hz-face.  Figure 3(a) shows five representative subjects. Because hemoglobin in blood absorbs red light, a pulse wave was calculated using the ratio between red light irradiation (660 *μ*m) and infrared light (940 *μ*m). The pulse frequency indicated by this apparatus is the average frequency of the previous eight pulses. The recordings were made as follows: during RS for 30 s; again during RS for 1 min; during 89 Hz-face stimulation for 2 min; after a 30 s rest, during 114 Hz-face stimulation for 2 min; finally, during RS for 30 s to 1 min. This experiment was performed between 3:00 and 5:00 pm in a quiet, temperature-controlled room.

### 2.4. HRV Analysis during Various Stimuli

HRV module analysis was used to measure R wave to R wave (RR) intervals (*n*_1_, *n*_2_, *n*_3_, *n*_4_, etc.) in ECG recordings. An example is shown in Figure 3(b). We recorded changes in the HRV (heart rate variability) of heart rate (HRV module, AD Instruments, Tokyo, Japan) during the RS, 89 Hz-face, Mozart, Mozart + 89 Hz-face, 89 Hz-neck, and Noise treatments. The HRV module data was produced using a period-histogram-analysis program and was processed by distributing the length of the RR interval. Typical values during various stimuli were assessed based on the mean peak values during the recording period. Heart rates during RS and various stimuli were recorded for 3 min and then the data were analyzed offline. We conducted these examinations on 18 normal subjects (12 males, 6 females; mean age, 25 years). This experiment was performed between 3:00 and 5:00 pm in a quiet, temperature-controlled room.

### 2.5. Pupillary Reflex

**Figure 4:**
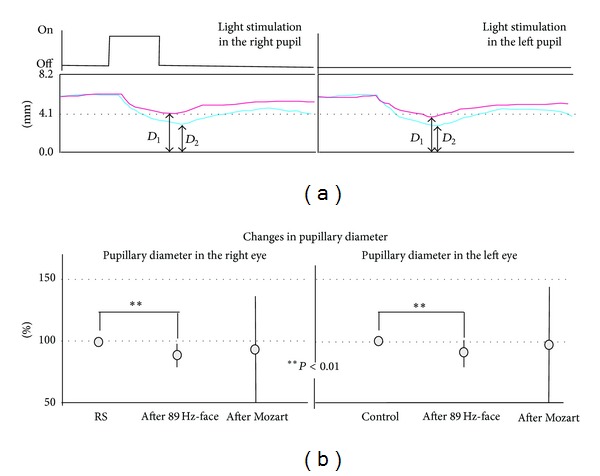
Effects of the pupillary reflex following the right-side light stimulation among the resting state (RS), 89 Hz-S on the face (89 Hz-face), and listing to Mozart (Mozart). (a) Typical example of data from the pupillary reflex. Note that the right pupillary reflex data after the light stimulation in right pupil evoked left pupillary reflex data after light stimulation in the left pupil. Red and blue waves showed the typical pupillary reflex after the RS and 89 Hz-face, respectively. (b) Changes in pupillary diameter in the right and left pupils after the RS, 89 Hz-face, and Mozart. We converted all values to the percentage of RS values (one hundred).

To explore changes in autonomic activity, we examined the transverse diameter of pupil constriction or dilation after vibrotactile stimulation, using the IRIS device (Iriscorder, Hamamatsu Photonics Co., Tokyo, Japan). This tool records the transverse diameter reaction and takes a picture of the eye while illuminating it with visible light (infrared radiation). The resulting image records the condition of the IRIS and eyeball movement on the monitor. For example, when normal subjects are exposed to continuous light stimulation for 1 s, a pupillogram such as that shown in Figure 4(a) is obtained; constricted pupils indicate parasympathetic activity, and dilated pupils indicate sympathetic activity. Pupil diameter in normal subjects is 2–5 mm, and it changes under various conditions. Pupil diameter was analyzed in this way during the presentation of various stimuli (RS, 89 Hz-face, and Mozart). The diameter of both eyes of all subjects was measured after a 3-minute rest or after presentation of the stimuli. Figure 4(a) shows the timeline of this experiment. The pupillary test is noninvasive and enables real-time diagnosis. We examined the initial diameter (*D*_1_) and the final diameter (*D*_2_) after 89 Hz-face stimulation. We conducted these examinations on eight normal subjects (six males, two females; mean age, 25 years). This experiment was performed between 3:00 and 5:00 pm in a quiet, temperature-controlled room.

### 2.6. fNIRS of the Frontal Cortex

**Figure 5:**
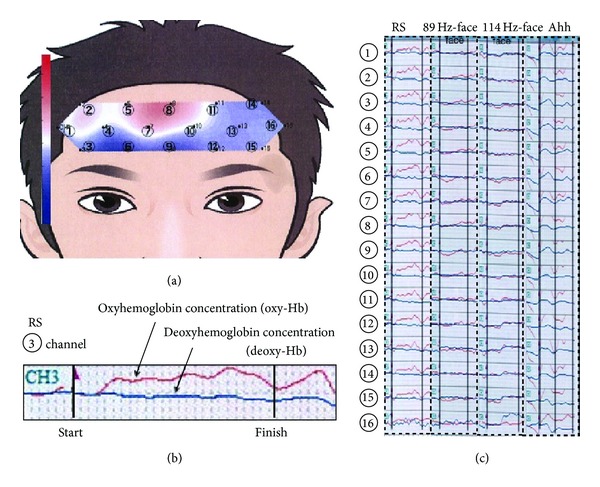
The fNIRS data recorded in the frontal cortex. (a) A computerized numerical analysis of variations in oxyhemoglobin (oxy-Hb) concentration during RS, 89 Hz-face, 114 Hz-face, and Ah~ (“Ah~” phonation). Red (plus) and blue shading (minus) indicate differences in frontal cortex activation, whereas the white band indicates nonactivation. Numbers indicated the positions of LED probes (16 channels). (b) Red and blue waves indicate the typical example of oxy-Hb and deoxy-Hb concentrations in the channel 3. The first vertical line indicates the start line of the various stimuli and the end vertical line indicates the finish line. (c) Changes in fNIRS activities during 2 min between solid lines of various vibrotactile stimuli and vocalizing “Ah~” sounds.

The fNIRS (functional near-infrared spectroscopy) recordings of the frontal cortex were made using a 16-channel fNIRS instrument (OEG16 instrument, Spectratech Inc., Shelton, CT, USA). The fNIRS probe assembly consisted of six light-emitting diodes (LEDs), each of which emitted two wavelengths (770 nm and 840 nm), and six photodiodes. The sources and detectors were arranged symmetrically in an area of 3.0 × 14.0 cm, with a nearest source-detector separation of 2.0 cm, and measurements were made at 16 points along the frontal cortex. A Velcro band held the probe assembly securely to the forehead of subjects during scanning and extended from ear to ear horizontally and from hairline to eyebrows vertically. Each LED was turned on in sequence and the diffuse NIR light from each source was acquired through the cortical region at the nearest detector. The sampling rate across all 16 channels was 0.76 Hz.Figure 5(a) shows the 16-channel computerized analysis, and the expanded waves are shown in Figure 5(b).

Furthermore, fNIRS oxy-Hb concentrations were measured for 2 min (between the start and finish lines shown in Figures 5(b) and 5(c)), under the following conditions: RS, 89 Hz-face, and 114 Hz-face, including pre- and poststimulation for 30 s. They were again measured as subjects said “Ahh” and then as they listened to classical music (Mozart, Eine kleine Nachtmusik) and as they listened to noise. These analyses were conducted on 27 normal subjects (20 males, 7 females; mean age, 22 years). As we found a larger standard deviation (SD) while subjects listened to Mozart, we thought it was important to determine whether the subjects were fans of Mozart or not. Thus, we divided the subjects into fans (7 subjects) and nonfans (20 subjects) of classical music. These experiments were performed between 3:00 and 5:00 pm in a quiet, temperature-controlled room.

### 2.7. Comparison and Analysis in Each Data

We want to investigate the relationship of the same subject of contrast between before and after. This is suitable for analysis by paired *t*-test. Furthermore, we are separated by examining of standard deviation (SD) of each data. In particular, the SD is a barometer of the extent of variation in data.

## 3. Results

### 3.1. Total Salivation during Presentation of Various Stimuli

We recorded amount of total salivation between before and after stimuli with the use of the cotton-roll method, as shown in Figure 1(c). Total salivation averaged 0.85 ± 0.38 (SD) mL in the RS, 1.11 ± 0.54 mL in 89 Hz-face, 1.12 ± 0.77 mL in Mozart, 0.87 ± 0.52 mL in Mozart + 89 Hz-face, 0.80 ± 0.40 mL in 89 Hz-neck, and 0.80 ± 0.46 mL in Noise. A significant difference was observed between RS and 89 Hz-face, between 89 Hz-face and 89 Hz-neck, and between 89 Hz-face and Noise (paired *t*-test *P* < 0.05, Figure 2). These results show that 89 Hz-face produced the most salivation (although Mozart had the highest average value, it also had the largest SD).

In addition, the daily use of vibrotactile stimulation did not lead to adaptation, and thus the continued use of this apparatus should not be a problem.

### 3.2. Pulse Frequency during Vibrotactile Stimuli

The pulse frequency data for five typical subjects are shown in Figure 3(a). All subjects showed a decrease in pulse frequency during 89 Hz-face compared to during 114 Hz-face. Five subjects showed a decrease during 114 Hz-face compared to RS, but the frequency during 89 Hz-face showed the largest decrease in all subjects (Figure 3(a)). These results indicate that 89 Hz-face elicited parasympathetic activity, causing the subject to feel relaxed. However, as changes in pulse, as shown in Figure 3(a), during each of the stimuli and RS were ambiguous, we also conducted HRV analysis, as detailed below and shown in Figures 3(b) and 3(c). Namely, in the analysis of heartbeat pulse analysis was unclear, but HRV analysis can categorize changes in heart rate.

### 3.3. Analysis of HRV during Various Stimuli

The HRV analysis results for the 10 subjects are shown in Figure 3(c). The values were 757.5 ± 57.0 ms for RS, 893.1 ± 189.5 ms for 89 Hz-face, 771.7 ± 86.7 ms for Mozart, 875.3 ± 188.3 ms for Mozart + 89 Hz-face, 901.7 ± 188.4 ms for 89 Hz-neck, and 831.7 ± 114.6 ms for Noise (Figure 3(c)). A significant difference was observed between RS and 89 Hz-face (paired *t*-test, *P* < 0.01), between RS and 89 Hz-neck (paired *t*-test, *P* < 0.01), and between RS and Noise (paired *t*-test, *P* < 0.05) (Figure 3(c)). Thus, 89 Hz-neck had the widest RR interval.

### 3.4. Pupillary Reflex

Of particular note, *D*_1_ (evoked by the pupil reflex after RS) and *D*_2_ (evoked by the pupil reflex after 89 Hz-face) represent a typical example: an absolute decrease between RS (red waves) and 89 Hz-face (blue waves) (Figure 4(a)).  Figure 4(b) shows the contraction percentages (*D*_2_/*D*_1_) after each stimulus. Significant differences were observed between the RS and 89 Hz-face (paired *t*-test: *P* < 0.01) in both eyes (Figure 4(b)). Although the average pupillary reflex after Mozart was small, the SD was large.

### 3.5. fNIRS of the Frontal Cortex

The device used for these recordings measures the concentration of hemoglobin in brain blood flow from 16 channels in the frontal cortex. Figure 5 shows a schema of the oxy-Hb (red wave) and deoxy-Hb (blue wave) concentrations evoked during the presentation of the various stimuli. Figure 5(c) shows an example of the waves in the fNIRS 16-channel recording. During RS, all channels showed increased oxy-Hb activity. However, during 114 Hz-face, channels 1, 13, 14, and 16 showed increased deoxy-Hb activity and channels 3, 6, 7, and 9 showed decreased oxy-Hb activity. Furthermore, during the pronunciation of “Ahh,” all channels showed increased oxy-Hb activity. During 89 Hz-face, all channels showed almost no activity.

**Figure 6:**
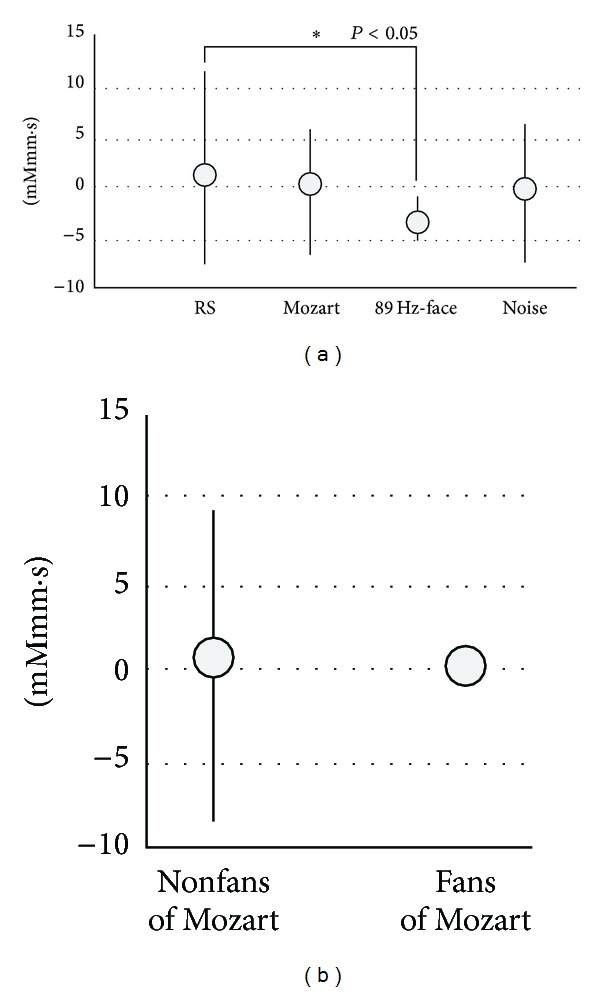
Integral values of oxy-Hb concentration produced with vibrotactile stimuli for 2 min are shown. Integral values were derived from oxy-Hb concentrations of channels 4, 7, 10, and 13 in the central part of the frontal cortex, as shown in Figure 4(a). (a) Integral values produced in the resting state (RS), listening to classical music (Mozart, Eine Kleine Nachitmusik), 89 Hz-S on the face (89 Hz-face), and listening to noise (Noise). There was a significant difference between RS and 89 Hz-face (paired *t*-test, *P* < 0.05). (b) Furthermore, we divided the Mozart listening group into fans of Mozart and no-fans of Mozart. Note that there were wide differences between the fans and nonfans of Mozart groups. Thus, nonfans group may listen as the noise, even if listening to Mozart.

Previous studies [5, 7] have reported a close relationship between local (or regional) cerebral blood flow (rCBF), in particular oxy-Hb, and the field potential in the somatosensory cortex in rats, in response to peripheral stimuli. Based on these reports, we computed integral rates of oxy-Hb over 2 min, as shown between the longitudinal bars of the recording waves (Figure 5(c)). In particular, we focused on changes in oxy-Hb in channels 4, 7, 10, and 13 in the central part of the frontal cortex, as shown in Figure 6(a). The value was 1.64 ± 7.46 mMmm·s during RS, 0.64 ± 6.46 mMmm·s during Mozart, −2.79 ± 2.12 mMmm·s during 89 Hz-face, and −0.15 ± 6.72 mMmm·s during Noise. “mMmm·s” indicated the value of integral in signal averaging.

A significant difference was observed between RS and 89 Hz-face (paired *t*-test *P* < 0.05). Furthermore, we found a large SD in the Mozart data, so we reran the analyses after dividing the Mozart listening group into fans (0.60 ± 0.55 mMmm·s) and nonfans (0.65 ± 9.42 mMmm·s) (Figure 6(b)). The difference became much less. Thus, if the SD in the fans is small, the measurement is reliable.

From this reason, the subjects who were not fans of Mozart may have been interpreting the music as noise.

## 4. Discussion

We made the vibrotactile apparatus to prevent contracture after the facial muscles surgery. However, many patients after the use of this apparatus complained of the increased salivation. Furthermore, in normal subjects, NIRS activity in the frontal cortex during the vibrotactile stimulation of 89 Hz-face showed the zero level of oxy-Hb and deoxy-Hb.

Are parasympathetic activities evoked by 89 Hz-face? When we are frightened, our heartbeat increases [9]. The parasympathetic nervous system is responsible for rest and digestion and for maintaining basal heart rate, respiration, metabolism, salivation, and contraction of pupillary diameter under normal or resting conditions [8–10]. We examined parasympathetic effects based on changes in heart rate (pulse frequency), pupillary reflex (diameter of the pupil), and salivary secretion during vibrotactile stimuli.

As shown in previous studies [1–3], vibrotactile stimulation and listening to classical music resulted in more salivation during 89 Hz-face and when listening to Mozart. However, listening to Mozart had the largest SD. Furthermore, the results between 89 Hz-face and RS, 89 Hz-neck, and also Noise were significant (Figure 2; paired *t*-test, *P* < 0.05). Namely, 89 Hz-face produced the most effective salivation result.

There were also changes in pulse frequency in normal subjects in response to 89 Hz-face and 114 Hz-face (Figure 3(a)), with a decrease during the former and an increase during the latter compared to that of 89 Hz-face. However, as Figure 3(a) shows an ill-defined frequency, we analyzed the ECG wave to also assess HRV [8] and found that 89 Hz-face and 89 Hz-neck had the biggest RR intervals (paired *t*-test, *P* < 0.01; Figures 3(b) and 3(c)). In particular, 89 Hz-face produced the most effective parasympathetic activity and the greatest salivation and HRV. The pupillary reflex contracts the pupil via parasympathetic activity [9]. Pupillary diameter in our experiments showed the greatest contraction after 89 Hz-face, as indicated from *D*_1_ to *D*_2_ in Figure 4(a). In particular, the pupils evoked by 89 Hz-face contracted the most compared to listening to Mozart (Figure 4(b)). Thus, 89 Hz-face resulted in lower pulse frequency, increased salivary secretion, and contracted pupils, suggesting that the stimulation activated the parasympathetic system (Figures 2–4). A relaxed feeling was produced in many subjects during 89 Hz-face; therefore, we believe that parasympathetic stimulation occurred as a result of 89 Hz-face.

In normal subjects, typical changes of NIRS parameters during neuronal activities show the increase of oxy-Hb and total-Hb and the decrease of deoxy-Hb. In particular, NIRS in the frontal cortex during language activities showed the increase of oxy-Hb and total-Hb and the decrease of deoxy-Hb. However, NIRS in the frontal cortex during videogaming showed the decrease of oxy-Hb and total-Hb and the increase of deoxy-Hb. Namely, NIRS activity in the frontal cortex during video gaming reported the inhibition of neuronal activities in the frontal cortex. Generally, NIRS activity pattern of oxy-Hb in the first motor cortex (*M*_1_) showed the increase during movements. The discrepancy of NIRS pattern between frontal cortex and *M*_1_ may be related to the difference of networks.

We find the appearance of zero level in NIRS (oxy-Hb and deoxy-Hb) of the frontal cortex during 89 Hz-face. Although the increase/decrease of oxy-Hb and deoxy-Hb was discussed by many reports, the appearance of the zero level of oxy-Hb and deoxy-Hb was unclear. Furthermore, the zero level of oxy-Hb and deoxy-Hb during the 89 Hz-face produced the increased salivation. In particular, we think that the phenomenon of the zero level may exist due to the parasympathetic activity. So, we intend to think about a mechanism for the zero level of NIRS.

However, the frontal cortex is associated with cognitive function, including memory, attention, abstract reasoning, and higher cognitive activity [11]. We recorded changes in the frontal cortex using fNIRS to examine typical changes in fNIRS parameters based on increased oxy-Hb and decreased deoxy-Hb, as shown in the RS and “Ahh” phonation treatments (Figure 5(c)). However, 89 Hz-face showed almost no activity in the two waves (oxy-Hb and deoxy-Hb waves: red and blue waves in Figure 5(b)) in all channels, although 114 Hz-face did increase deoxy-Hb in some channels (Figure 5(c)). Many reports of fNIRS activity have focused on excitatory behavior to increase oxy-Hb, but no reports are available on increased deoxy-Hb [5, 7, 12, 13]. Animal experiments have shown that changes in oxy-Hb and fNIRS data are related, and activity changes in oxy-Hb are used as a neuronal activity index [13]. Furthermore, previous studies [5, 7] have reported a close relationship between values of rCBF and the field potential in the somatosensory cortex of rats, in response to peripheral stimuli. We also found the same result when stimulating an awake cat's whiskers as shown by increased Oxy-Hb activity in the cat's somatosensory cerebral cortex [12]. Thus, oxy-Hb has a tight connection with neuronal activity, in particular when we have a clear sense of perception and sympathetic nerves are activated when we are excited. Furthermore, we thought that waves of increased or decreased oxy-Hb concentrations might be associated with sympathetic activity. The changes in oxy-Hb produced by 89 Hz-face on the parotid and submandibular glands may indicate mental stability. Increased activity patterns in the frontal cortex are associated with speech and decreased patterns are associated with playing TV games [14]. The responses we observed may have been influenced by mental stability and excitability because they indicate control of sophisticated mental functions that are produced by complex networks. Because brain activity in the frontal cortex increases when subjects are speaking and decreases when they play TV games [14], we suggest that the phenomenon has a profound effect on the parasympathetic or sympathetic activity. Specifically, we suggest that such brain activity during conscious speaking is associated with a sympathetic effect and that playing a TV game is associated with responsive movement, under a nonsympathetic effect. We focused on fNIRS waves in channels 4, 7, 10, and 13 in the central part of the frontal cortex. The near-zero levels of oxy-Hb and deoxy-Hb that we detected may have been due to the same tendency in oxy-Hb concentration between 89 Hz-face and subjects who liked to listen to classical music (Figures 5(c) and 6(b)). Thus, we suggest that the phenomenon evoked by 89 Hz-face was produced by excitation of the parasympathetic system. Although 89 Hz-face always caused parasympathetic excitation, listening to classical music resulted in different activity depending on music preference (Figures 6(a) and 6(b)), because subjects who enjoy classical music might find listening to it relaxing, whereas those who dislike it might perceive it as noise. These findings suggest that the effects caused by 89 Hz-face and the feeling sensed by those listening to Mozart who enjoyed it may be the same. Thus, we suggest that the feelings evoked by 89 Hz-face were produced by parasympathetic activity. Furthermore, activity in the frontal cortex may indicate autonomic activity.

## 5. Conclusions

The parasympathetic nervous system is responsible for rest and digestion as well as maintaining basal heart rate, respiration, metabolism, salivation, and contraction of pupillary diameter, among other roles, under normal and resting conditions [8–10]. We examined parasympathetic effects based on changes in heart rate (pulse frequency), pupillary reflex (diameter of pupils), and salivary secretion during vibrotactile stimuli. The findings suggest that the effects caused by 89 Hz-face and the feeling sensed by those listening to Mozart who enjoyed it may be the same. Thus, we suggest that the feelings evoked by 89 Hz-face were produced by parasympathetic activity. Furthermore, activity in the frontal cortex may indicate autonomic activity.

## Abbreviations

HRV: Heart rate variability module
fNIRS: Functional near-infrared spectroscopy
rCBF: Regional cerebral blood flow
oxy-Hb: Oxyhemoglobin
deoxy-Hb: Deoxyhemoglobin.
